# Readability of state-sponsored advance directive forms in the United States: a cross sectional study

**DOI:** 10.1186/1472-6939-11-6

**Published:** 2010-04-25

**Authors:** Luke A Mueller, Kevin I Reid, Paul S Mueller

**Affiliations:** 1 Department of Dental Specialties, Mayo Clinic, Rochester, Minnesota, USA; 2 Division of General Internal Medicine, Mayo Clinic, 200 First Street SW, Rochester, Minnesota, USA 55905

## Abstract

**Background:**

State governments provide preprinted advance directive forms to the general public. However, many adults in the United States (US) lack the skills necessary to read and comprehend health care-related materials. In this study, we sought to determine the readability of state government-sponsored advance directive forms.

**Methods:**

A cross sectional study design was used. The readability of advance directive forms available online from all 50 US states and the District of Columbia was determined using 6 validated readability scales.

**Results:**

Overall, 62 advance directive forms were obtained. For 47 states, forms were available by way of government-sponsored Web sites. The average (SD) readability (with the Flesch-Kincaid score) of all forms was grade level 11.9 (2.6). Similar results were obtained with the other readability scales. No form had a readability score at the 5th grade level or lower, the level recommended by the National Work Group on Literacy and Health. The readability of the forms exceeded this level by an average of 6.9 grade levels (95% confidence interval, 6.3-7.6; *P *< .001). Only 5 of the forms had a readability score at 8th grade level or lower, the average reading skill level of US adults. The readability of the forms exceeded this level by an average of 3.9 grade levels (95% confidence interval, 3.3-4.6; *P *< .001).

**Conclusions:**

The readability of US state government-sponsored advance directive forms exceeds the readability level recommended by the National Work Group on Literacy and Health and the average reading skill level of most US adults. Such forms may inhibit advance care planning and therefore patient autonomy.

## Background

Advance care planning promotes patient autonomy. It is a process in which patients, working with their clinicians, other members of their health care team, and their loved ones, articulate their goals and preferences about future health care decisions in the event they cannot speak for themselves (eg, life-sustaining treatments at the end of life). This process includes completion of an advance directive (AD). The most common types of ADs are 1) the health care power of attorney, in which the patient designates another person for making future health care decisions; 2) the living will, in which the patient lists instructions about future treatments; and 3) the combined AD, which has features of both a health care power of attorney and a living will [1]. Many people who complete an AD use preprinted forms produced by state governments, health care institutions, and other groups. These forms are widely available at health care institutions and online.

Generally, adults in the United States (US) view ADs favorably. Nevertheless, only about 20% of adults in the United States have completed an AD. One patient characteristic associated with AD completion is education level. In 1 study, AD use was highest among college graduates and lowest among persons with less than 8 years of formal education [2]. In another study, more than 90% of patients who completed an AD had at least a high school diploma [1]. In a study of elders (age, ≥ 65 years), those who had completed an AD were highly educated and did not consider the AD form too long, whereas those who had not completed an AD--but wanted to--described family issues and the need for assistance as barriers to completing the form [3].

The association between education level and AD completion is likely mediated by health literacy. Health literacy is "a constellation of skills, including the ability to perform basic reading and numerical tasks required to function in the health care environment" [4]. It is lowest among patients with low education levels. Health literacy is also low among elders, poor persons, ethnic minority populations, and recent immigrants [5,6]. Low literacy is associated with fewer health-promoting behaviors, poorer health status, and higher rates of hospitalization and health care costs [5].

Notably, the average reading skill of US adults is at the level of 8th grade or lower; in other words, 90 million adults have reading skills below the high school level [5,7]. Of these adults, many lack the minimum skills necessary for comprehending patient education materials, informed consent forms, medication labels, and other health care-related materials [6]. Indeed, hundreds of studies have shown that health care-related materials, regardless of the topic or content, are incomprehensible to most US adults [7-11]. For example, most patient education materials [4,7] and most consent forms [9] are written at the 10th grade level or higher. Because of these findings, the National Work Group on Literacy and Health recommends a 5th grade readability level for health care-related written materials [7].

We report the results of an analysis of the readability of state government-sponsored AD forms from all 50 states and the District of Columbia in the United States. We analyzed these forms because laws regarding ADs differ among the states and individuals interested in completing ADs might look first to their own state governments as a source for legally valid forms. We hypothesized that the readability of these forms exceeded the readability level recommended by the National Work Group on Literacy and Health and the average reading skill level of US adults.

## Methods

Nearly all AD forms in our study were obtained from publicly accessible, state government-sponsored Web sites. However, several states and the District of Columbia did not provide AD forms on their Web site. For these states, we used the Google search engine to locate and obtain AD forms. The search terms *advance directive*, *living will*, and *power of attorney for health care *were used, along with the state's name. The top Web sites identified by the search engine were examined. The AD forms on these Web sites that met the legal criteria for the respective jurisdictions were used. Additional file 1 lists the links to the Web sites used to obtain the AD forms, organized by state and the District of Columbia. The sites were accessed between February 1, 2009, and July 15, 2009.

Many AD forms were available as Microsoft Word electronic files. The other AD forms were available as portable document format files. The files were converted into Word files using a converter software package (Nuance PDF Converter 5; Nuance Communications, Inc, Burlington, Massachusetts). These converted files were checked manually to determine whether the conversions were accurate.

Next, each AD form was subjected to readability analysis using the software package Readability Studio for Windows (Oleander Software, Ltd, Vandalia, Ohio). For each analysis, 6 readability scales were used--Flesch-Kincaid, Automated Readability Index, Linsear Write, New Fog Count, Simplified Automated Readability Index, and Flesch Reading Ease--to determine the readability of the text by analyzing syllable count, length of sentences, complexity of words, and other text characteristics. Each scale except the Flesch Reading Ease scale identifies a reading grade level between 0 and 19.0 for the text analyzed and uses a scale in which the lower the scale score, the easier the text to read. For example, a score of 11.0 indicates that the sample text is written at a reading level of 11th grade. Notably, the 6 scales differ in how they determine grade levels of text:

Flesch-Kincaid--Influenced by sentence length and syllable count. Shorter sentences and less complex words lower the score.

Automated Readability Index--Influenced by sentence length and character count. Shorter sentences and shorter words lower the score.

Linsear Write--Influenced by sentence length and words containing 3 or more syllables. Shorter sentences and less complex words of only 1 or 2 syllables lower the score.

New Fog Count--A modified version of the Gunning Fog Index and influenced by words containing 3 or more syllables. Less complex words lower the score.

Simplified Automated Readability Index--A modified version of the Automated Readability Index and influenced by sentence length and character count. Shorter sentences and words lower the score.

Flesch Reading Ease--Influenced by sentence length and syllable count. It does not identify a reading grade level. Instead, it provides a score between 0 and 100, and the higher the score, the easier the read. Use of shorter sentences and less complex words increase the score.

One-sample *t *tests were used to compare the mean readability scores of the AD forms as determined by the Flesch-Kincaid readability scale with the recommended readability level of the National Work Group on Literacy and Health and the average reading skill level of US adults. The Flesch-Kincaid scale was used for this purpose because it is the most widely used readability scale and is reliable and valid [9]. All analyses were conducted using a statistical software package (JMP 8; SAS Institute Inc, Cary, North Carolina). The Mayo Clinic Institutional Review Board determined that this study did not constitute research involving human subjects as defined under 45 CFR 46.102.

## Results

In total, 62 AD forms from the 50 states and the District of Columbia were obtained. For 47 states, forms were obtained from state government-sponsored Web sites. For 3 states and the District of Columbia, forms were obtained using the Google search engine. Some Web sites had more than 1 AD form (eg, a health care power of attorney form and a living will form), whereas most sites had 1 combined AD form (Additional file 1).

**Figure 1:**
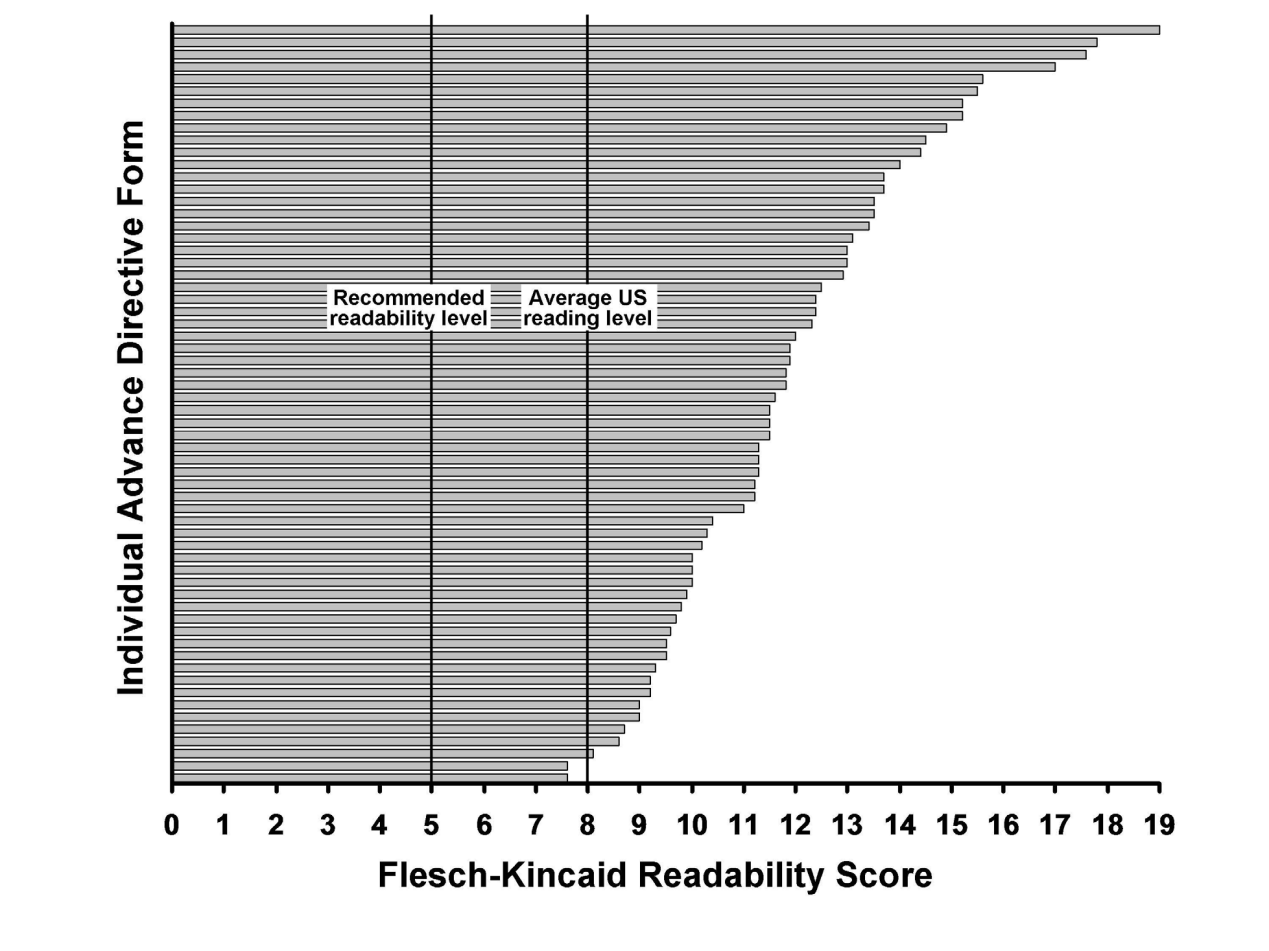
**Flesch-Kincaid readability scores of advance directive forms of the 50 states and the District of Columbia (DC) of the United States**. Each bar represents grade level readability of 1 of 62 forms (mean [range], 11.6 [7.6-19.0]).

The median page length was 5.5 pages (range, 1-41 pages). The readability scores of the 62 AD forms, as determined by the Flesch-Kincaid scale, are listed in Additional file 1 and displayed in the Figure 1. None of the AD forms had a readability score at the 5th grade reading level or lower, and only 5 forms had readability scores at the 8th grade level or lower.

Aggregate results of the readability analyses using the 6 different readability scales are shown in Table 1. For example, with the Flesch-Kincaid readability scale, the 62 AD forms had a mean score of grade level 11.9 (SD, 2.6) and the median score was grade level 11.6 (range, 7.6-19.0). Similar results were obtained using the other 5 readability scales.

**Table 1:** Median and mean readability scores of advance directive forms for the 50 states and the District of Columbia of the United States using 6 different readability scales.

Scale	Median (range)	Mean (SD)
**Flesch-Kincaid**	11.6 (7.6 to 19)	11.9 (2.6)
**ARI**	11.3 (6.5 to 19)	11.5 (3.0)
**Linsear Write**	13.4 (4.1 to 19)	13.5 (3.6)
**Few Fog Count**	11.9 (3.6 to 19)	12.3 (3.6)
**SARI**	9.5 (5.1 to 19)	9.8 (2.8)
**FRESV**	47.5 (0 to 62)	45.5 (11.3)

Abbreviations: ARI, Automated Readability Index; FRESV, Flesch Reading Ease scale value; SARI, Simplified Automated Readability Index.

The average readability score of the AD forms exceeded the grade level recommended by the National Work Group on Literacy and Health by an average of 6.9 grade levels (95% confidence interval [CI], 6.3-7.6; *P *< .001). It also exceeded the average reading skill level of US adults by an average of 3.9 grade levels (95% CI, 3.3-4.6; *P *< .001). Only 22 AD forms had scores at the 10th grade level or lower; the average score of the AD forms exceeded this level by an average of 1.9 grade levels (95% CI, 1.3-2.6; *P *< .001). These results were unchanged when the AD forms acquired from only the 47 state government-sponsored Web sites were used.

Samples of text from the AD forms of 3 states are provided in Table 2. These samples show the wide range of readability among the AD forms analyzed in this study.

**Table 2:** Samples of text taken from advance directive forms from Oregon, Delaware, and Utah, showing various readability levels.

State	Power of Attorney	Living Will
Oregon (F-K score, 7.6)	I appoint ____ as my health care representative.	Close to Death. If I am close to death and life support would only postpone that moment of my death: A. INITIAL ONE: ____I want to receive tube feeding. ____I want tube feeding only as my physician recommends. ____I DO NOT WANT tube feeding. B. INITIAL ONE: ____I want any other life support that may apply. ____I want life support only as my physician recommends. ____I want NO life support.

Delaware (F-K score, 11.8)	I designate ____ as my agent to make health care decisions for me. If he/she is not living, willing or able, or reasonably available, to make health care decisions for me, then I designate ____ as my agent to make health care decisions for me.	I do not want my life to be prolonged if (please check all that apply) ____(i) I have a terminal condition (an incurable condition from which there is no reasonable medical expectation of recovery and which will cause my death, regardless of the use of life-sustaining treatment). In this case, I give the specific directions indicated: (Individuals check columns labeled "I want used" or "I do not want used" next to the following: "Artificial nutrition through a conduit," "Hydration through a conduit," "Cardiopulmonary resuscitation," "Mechanical respiration," and "Other [explain]").

Utah (F-K score, 17.8 [POA] and 19 [living will])	I, ____ ... being of sound mind, willfully and voluntarily appoint ___ ... as my agent and attorney-in-fact, without substitution, with lawful authority to execute a directive on my behalf under Section 75-2-1105, governing the care and treatment to be administered to or withheld from me at any time after I incur an injury, disease, or illness which renders me unable to give current directions to attending physicians and other providers of medical services.	I declare that if at any time I should have an injury, disease, or illness, which is certified in writing to be a terminal condition or persistent vegetative state by two physicians who have personally examined me, and in the opinion of those physicians the application of life sustaining procedures would serve only to unnaturally prolong the moment of my death and to unnaturally postpone or prolong the dying process, I direct that these procedures be withheld or withdrawn and my death be permitted to occur naturally.

Abbreviations: F-K, Flesch-Kincaid; POA, power of attorney

## Discussion

We found that AD forms, nearly all of which were acquired from state government-sponsored Web sites, are complex documents with high grade level readability scores. All of the AD forms exceeded the recommended readability level of the National Work Group on Literacy and Health, nearly all forms exceeded the average reading skill of US adults, and most forms exceeded the 10th grade reading skill level. These findings are consistent with those of a prior study, published in 1997, on the readability of a convenience sample of 10 AD forms derived from various government and nongovernment sources [12] and of hundreds of studies on the readability of materials written for patients [5,8].

Our findings are important for several reasons. First, the completion of an AD promotes patient autonomy by allowing a patient to articulate in advance his or her goals and preferences for future care in the event that the patient lacks decision-making capacity. Providing incomprehensible AD forms to patients with low reading skills does not promote their autonomy; incomprehensible AD forms restrict patient autonomy by inhibiting patients from expressing their goals and preferences for future health care. Second, the Patient Self-Determination Act requires that hospitals provide information to patients about their rights related to and state laws and requirements regarding ADs [13]. However, the intent of the Patient Self-Determination Act is not that patients simply be given written materials about ADs (eg, brochures, forms) but that they understand the information and its relevance. Third, state governments are responsible for both protecting the rights and promoting the welfare of their citizens. Governments fail in these duties when they provide incomprehensible AD forms to their citizens. Fourth, the Joint Commission requires that hospitals establish a mechanism to determine whether their informed consent procedures, medication, and discharge instructions and other communications are understood by patients [3]. Presumably, this requirement also pertains to AD forms. Nevertheless, providing AD forms that do not exceed the literacy levels of most US adults is possible. A recent randomized trial showed that an AD form written at the 5th grade reading level was rated by study participants--especially those with low literacy--as easier to use and more useful in advance care planning than a state government-sponsored (California) AD form written at the 12th grade level. Furthermore, significantly more participants randomized to the easier-to-read form successfully completed an AD [14].

Although none of the 62 AD forms achieved the recommended readability level of 5th grade, we nonetheless observed wide variability in the readability of the forms by jurisdiction. For example, the Flesch-Kincaid readability score for Oregon's combined AD form was grade level 7.6, whereas the readability score for Utah's living will and power of attorney forms were grade levels 19.0 and 17.8, respectively. Review of text samples taken from these AD forms affirms this wide variability (Table 2). Given the absence of a federal policy on the readability of AD forms and that each state and the District of Columbia have the authority to establish their own laws and requirements related to ADs, it is not surprising that the readability of these forms varies by jurisdiction [5].

Our study has several strengths. We obtained and analyzed AD forms from all 50 states and the District of Columbia, lending credibility to the conclusion that these jurisdictions do not provide forms that are readable to most of their citizens. In addition, we analyzed the entire text of the AD forms, not just samples of text from them. Finally, we used 6 readability scales in our analyses, including the reliable, valid, and frequently used Flesch-Kincaid scale [9].

Our study also has several limitations. It is possible that some state governments provide AD forms that are easy to read but are not available online. Also, some patients may find AD forms that are easy to read at nongovernment Web sites [15,16] or from non-Web resources. Notably, one of these forms ("Five Wishes" [16]), which is widely used and advertised as "written in everyday language," has a Flesch-Kincaid scale score of 8.3. This form, while popular, does not meet the literacy level of many US adults. In addition, we did not evaluate the formatting characteristics of the forms (eg, font size, color schemes, illustrations), which might affect readability [9]. Finally, readability formulas do not ensure that written materials are understandable. For example, longer sentences and words, which would increase a readability score, might actually be more comprehensible to persons with low reading skills. Likewise, shorter sentences that use arcane words would lower a readability score yet may be harder to read and understand. Hence, authors of health care-related materials for patients should not rely solely on readability scales to ensure that text is comprehensible to patients [8].

What should be the response to our findings? First, clinicians should ask patients whether they have completed an AD. If not, clinicians should attempt to discern why and be mindful that patient education level and literacy may be factors inhibiting AD completion. If those are the inhibiting factors, the patients should be referred to facilitators (eg, social workers) who can assist with their completing the AD [1]. Second, state governments, health care institutions, and other organizations that develop AD forms and make them available to the general public should ensure that the forms are readable and understandable by most people. The 5th grade readability level recommended by the National Work Group on Literacy and Health is an appropriate goal for AD forms intended for the general public. However, clinicians, policy makers, and others who develop and make available AD forms should bear in mind that even a 5th grade readability level will be too difficult for many adult patients. For these patients, lower readability levels can be achieved by using narrative, dialogue, or video formats to present information related to ADs [7]. Overall, the most effective AD allows the patient to name a health care agent and express his or her goals and preferences for future health care, is detailed yet easy to use, and is disease specific, if appropriate [17].

## Conclusions

The readability of state government-sponsored AD forms significantly exceeds the readability level recommended by the National Work Group on Literacy and Health and the average reading skill level of most US adults. Such forms inhibit advance care planning and therefore patient autonomy. Governments, health care institutions, and other organizations that develop AD forms should ensure that the forms are readable and understandable by most people.

## Abbreviations

The following abbreviations were used in the text of this article: AD: advance directive; US: United States; CI: confidence interval.

## Competing interests

PSM is a member of the Boston Scientific Patient Safety Advisory Board and is an Associate Editor for *Journal Watch*. Neither relationship is directly related to the content of this paper. The other authors have no competing interest to report.

## Authors' contributions

LAM was the primary author of this paper. LAM participated in the study conception and design, data collection, coordination, analysis, and manuscript writing and revisions. KIR participated in the study conception and design, data collection, coordination, analysis, and manuscript revisions. PSM participated in the study conception and design, data collection, coordination, analysis, and manuscript writing and revisions. All authors read the paper for intellectual content and approved the final version of the manuscript.

## Pre-publication history

The pre-publication history for this paper can be accessed here:

http://www.biomedcentral.com/1472-6939/11/6/prepub

## Supplementary Material

Additional file 1 **Web links, number of pages, and the Flesch-Kincaid readability scale scores for advance directive forms from the 50 states and the District of Columbia (DC) of the United States**. This file contains Web links, dates accessibility verified, number of pages, and the Flesch-Kincaid readability scale scores for 62 advance directive forms from the 50 states and the District of Columbia (DC) of the United States. Click here for file
