# Supplementary material for: Readability of state-sponsored advance directive forms in the United States: a cross sectional study
Source: BMC Med Ethics. 2010 Apr 25;11:6. doi: 10.1186/1472-6939-11-6 (PMC2868033; doi:10.1186/1472-6939-11-6)
Supplement: Additional file 1 — Web links, number of pages, and the Flesch-Kincaid readability scale scores for advance directive forms from the 50 states and the District of Columbia (DC) of the United States. This file contains Web links, dates accessibility verified, number of pages, and the Flesch-Kincaid readability scale scores for 62 advance directive forms from the 50 states and the District of Columbia (DC) of the United States. [file 1472-6939-11-6-S1.DOC]

**Additional file 1.** Web links, dates accessibility verified, number of pages, and the Flesch-Kincaid readability scale score for 62 advance directive forms from the 50 states and the District of Columbia (DC) of the United States.

| **State or DC** | **Web Link** | **No. of**  **Pages** | **Flesch-Kincaid Score** |
| --- | --- | --- | --- |
|  |  |  |  |
| Alabama | http://www.alabar.org/members/consumer-guide_forms_8_2008.pdf  By way of http://www.medicaid.alabama.gov/resources/advance_directives.aspx?tab=5 (accessibility verified July 14, 2009) | 5 | 7.6 |
| Alaska | http://www.hss.state.ak.us/pdf/advancedirective.pdf (accessibility verified February 8, 2009) | 12 | 12 |
| Arizona | http://www.azsos.gov/Adv_Dir/HCD/forms.htm (accessibility verified February 8, 2009) | 2 | 9.2 |
| Arkansas^a^ | http://www.arkbar.com/whats_new/Advance%20Directive%20Model%20Form%20for%20ABA.pdf (accessibility verified February 8, 2009) | 4 | 13.1 |
| California | http://www.ag.ca.gov/consumers/pdf/AHCDS1.pdf (accessibility verified February 8, 2009) | 6 | 11.3 |
| Colorado^a^ | http://www.caringinfo.org/userfiles/File/Colorado.pdf (accessibility verified February 8, 2009) | 20 | 12.9 |
| Connecticut | http://www.ct.gov/ag/lib/ag/health/Adv_Directives_Combined_Form.pdf (accessibility verified February 8, 2009) | 4 | 11.6 |
| Delaware | http://www.dhss.delaware.gov/dhss/dsaapd/files/advancedirective.pdf (accessibility verified February 8, 2009) | 6 | 11.8 |
| DC^a^ | http://www.caringinfo.org/userfiles/File/DistrictofColumbia.pdf (accessibility verified May 22, 2009) | 23 | 8.7 |
| Florida | http://www.ahca.myflorida.com/MCHQ/Health_Facility_Regulation/HC_Advance_Directives (accessibility verified February 8, 2009) | 9 | 12.4 |
| Georgia | http://www.aging.dhr.georgia.gov/DHR-DAS/Georgia%20ADVANCE%20DIRECTIVE%20FOR%20HEALTH%2CARE-07.pdf (accessibility verified July 14, 2009) | 26 | 12.3 |
| Hawaii | http://www.hawaii.gov/health/eoa/Docs/AHCD.pdf (accessibility verified February 8, 2009) | 4 | 9.7 |
| Idaho | http://www2.state.id.us/ag/living_wills/LivingWill_DurablePowerOfAttorney.pdf (accessibility verified February 8, 2009) | 7 | 13 |
| Illinois | http://www.idph.state.il.us/public/books/Livin.PDF (accessibility verified February 8, 2009)  http://www.idph.state.il.us/public/books/PwrOf.PDF (accessibility verified February 8, 2009) | 1  3 | 14.5  14.9 |
| Indiana^a^ | http://www.caringinfo.org/userfiles/File/Indiana.pdf (accessibility verified February 10, 2009) | 20 | 13.5 |
| Iowa | http://www.state.ia.us/elderaffairs/Documents/Publications/GiftofPeaceofMind.pdf (accessibility verified February 10, 2009) | 22 | 10 |
| Kansas | http://www.agingkansas.org/Publications/resource_guide/kanpoa_health.pdf  By way of http://kansas.google.nicusa.com/search?q=power+of+attorney+for+health+care&entqr=0&output=xml_no_dtd&sort=date%3AD%3AL%3Ad1&entsp=0&client=kansas&ud=1&oe=UTF-8&ie=UTF-8&proxystylesheet=kansas&site=kansas (accessibility verified February 10, 2009) | 3 | 15.2 |
| Kentucky | http://ag.ky.gov/NR/rdonlyres/2DA643B3-B474-44B6-8A7E-9EDC7B88FD1C/0/living_will_packet.pdf (accessibility verified February 10, 2009) | 8 | 11.2 |
| Louisiana | http://www.sos.louisiana.gov/Portals/0/publications/pdf/Liv_Will_Dec_form.pdf (accessibility verified February 8, 2009) | 1 | 14.2 |
| Maine | http://www..themha.org/issues/advdirectivesform.pdf  By way of http://www.maine.gov/ag/elder_issues/living_will.shtml (accessibility verified July 14, 2009) | 14 | 9.5 |
| Maryland | http://www.oag.state.md.us/Healthpol/adirective.pdf (accessibility verified February 8, 2009) | 17 | 9 |
| Massachusetts | http://www.massmed.org/AM/Template.cfm?Section=Search&TEMPLATE=/CM/ContentDisplay.cfm&CONTENTID=2570 (accessibility verified February 10, 2009) | 4 | 12 |
| Michigan | http://www.michigan.gov/documents/mdch/mdch_AdvanceDirectivesPamphlet_196639_7.doc (accessibility verified July 14, 2009) | 41 | 10.3 |
| Minnesota | http://www.mnaging.org/pdf/MSS103-F.pdf (accessibility verified February 10, 2009) | 4 | 8.1 |
| Mississippi | http://www.msdh.state.ms.us/msdhsite/index.cfm/42,75,210,pdf/AdvanceDirectives%2Epdf (accessibility verified February 8, 2009) | 17 | 11.3 |
| Missouri | http://www.ago.mo.gov/publications/lifechoices/powerofattorney.pdf (accessibility verified February 8, 2009)  http://www.ago.mo.gov/publications/lifechoices/healthcarechoices.pdf (accessibility verified February 8, 2009) | 6  6 | 11.5  11.5 |
| Montana | http://www.doj.mt.gov/consumer/consumer/forms/advancedirective.doc (accessibility verified February 8, 2009) | 4 | 9.6 |
| Nebraska | http://www.hhs.state.ne.us/ags/docs/LivingWill.pdf (accessibility verified February 8, 2009)  http://www.hhs.state.ne.us/ags/docs/Power-of-Attorney.pdf (accessibility verified February 8, 2009) | 1  3 | 13.5  9.9 |
| Nevada | http://www.dhcfp.state.nv.us/HIPAA/NV%20Law%20Concerning%20Advanced%20Directives.pdf (accessibility verified February 8, 2009) | 14 | 11.2 |
| New Hampshire | http://www.gencourt.state.nh.us/rsa/html/x/137-j/137-j-mrg.htm (accessibility verified February 10, 2009) | 23 | 11.8 |
| New Jersey | http://www.state.nj.us/health/healthfacilities/documents/Itc/advance_directives.pdf (accessibility verified February 10, 2009) | 27 | 12.4 |
| New Mexico | http://www.nmaging.state.nm.us/pdf_files/AHCDforms.pdf  By way of http://www.newmexico.gov/ (accessibility verified July 14, 2009) | 4 | 11.9 |
| New York | http://www.cqcapd.state.ny.us/hottopics/advdifm.htm (accessibility verified February 10, 2009) | 8 | 10 |
| North Carolina | http://www.carolinasendoflifecare.org/pdf/NCLivingWill.pdf (accessibility verified July 14, 2009)  http://www.carolinasendoflifecare.org/pdf/ncPOA.pdf (accessibility verified July 14, 2009) | 5  7 | 11  13.7 |
| North Dakota | http://www.nd.gov/dhs/info/pubs/docs/aging/aging-healthcare-directives-guide.pdf (accessibility verified February 24, 2009) | 27 | 10 |
| Ohio | http://www.olrs.ohio.gov/other/OLRSPOA.pdf (accessibility verified February 8, 2009) | 12 | 13.7 |
| Oklahoma | http://okpalliative.nursing.ouhsc.edu/documents/AdirectiveHealthCare%20Form.pdf (accessibility verified February 24, 2009) | 4 | 15.5 |
| Oregon | http://egov.oregon.gov/DCBS/SHIBA/docs/advance_directive_form.pdf (accessibility verified February 8, 2009) | 7 | 7.6 |
| Pennsylvania | http://www.aging.state.pa.us/aging/lib/aging/Advance_Directives_brochure1.pdf (accessibility verified February 8, 2009) | 6 | 11.3 |
| Rhode Island | http://www.health.ri.gov/hsr/directives.php (accessibility verified July 14, 2009)  This 1 site has 2 forms:  Living Will  Durable Power of Attorney for Health Care | 2  12 | 10.4  9.8 |
| South Carolina | http://www.state.sc.us/dmh/804-97.htm (accessibility verified February 8, 2009) | 22 | 8.7 |
| South Dakota | http://www.sdbar.org/pamphlets/Living_Will.html (accessibility verified February 17, 2009) | 3 | 10.3 |
| Tennessee | http://health.state.tn.us/AdvanceDirectives/Advance_Care_Plan.pdf (accessibility verified February 17, 2009)  http://health.state.tn.us/AdvanceDirectives/Health_Care_Agent.pdf (accessibility verified February 17, 2009)  http://health.state.tn.us/AdvanceDirectives/Surrogate.pdf (accessibility verified February 17, 2009) | 2  1  1 | 8.6  9  9.3 |
| Texas | http://www.dads.state.tx.us/news_info/publications/handbooks/LivingWill-English.pdf (accessibility verified February 8, 2009)  http://www.dads.state.tx.us/news_info/publications/handbooks/MEDPOA-English.pdf (accessibility verified February 8, 2009) | 3  4 | 14  10.2 |
| Utah | http://www.hsdaas.utah.gov/pdf/Utah%20Living%20Will%20Form.pdf (accessibility verified February 8, 2009)  http://www.hsdaas.utah.gov/pdf/Utah%20Power%20of%20Attorney%20for%20Health%20Care.pdf (accessibility verified February 8, 2009) | 2  1 | 19  17.8 |
| Vermont | http://healthvermont.gov/regs/ad/AD_attachmentA.pdf (accessibility verified May 24, 2009) | 24 | 8.7 |
| Virginia | http://www.vda.virginia.gov/pdfdocs/AdvMedDir.pdf (accessibility verified February 17, 2009) | 2 | 17 |
| Washington | http://www.doh.wa.gov/livingwill/forms/HealthCareDirective.pdf (accessibility verified May 24, 2009)  http://www.doh.wa.gov/livingwill/forms/DurablePowerOfAttorneyForHealthCare.pdf (accessibility verified May 24, 2009) | 2  3 | 17.6  13.4 |
| West Virginia | http://www.wv.gov/Pages/Search.aspx?q=living%20will (accessibility verified May 2, 2009) | 1 | 13.1 |
| Wisconsin | http://dhs.wisconsin.gov/forms/AdvDirectives/F00060.pdf (accessibility verified July 14, 2009)  http://dhs.wisconsin.gov/forms/AdvDirectives/F00085.pdf (accessibility verified July 14, 2009) | 4  8 | 14.4  12.6 |
| Wyoming | http://search.wy.gov/search?q=cache:dlqJ9wIauXIJ:wdh.state.wy.us/Media.aspx%3FmediaId%3D2699+advance+directive&access=p&output=xml_no_dtd&site=default_collection&ie=UTF-8&client=default_frontend&proxystylesheet=default_frontend&oe=UTF-8 (accessibility verified May 24, 2009) | 7 | 11 |
|  |  |  |  |

^a^Advance directive forms for these 3 states and DC were not found through state government–sponsored Web sites. Instead, their advance directive forms were found using the Google search engine; sites listed first were used.
